# An “off-on” fluorescent nanosensor for the detection of cadmium ions based on APDC-etched CdTe/CdS/SiO_2_ quantum dots

**DOI:** 10.1016/j.heliyon.2024.e26980

**Published:** 2024-02-27

**Authors:** Jiaqian Chen, Haimei Meng, Zhijia Fang, Iddrisu Lukman, Jialong Gao, Jianmeng Liao, Qi Deng, Lijun Sun, Ravi Gooneratne

**Affiliations:** a College of Food Science and Technology, Guangdong Provincial Key Laboratory of Aquatic Product Processing and Safety, Guangdong Provincial Engineering Technology, Research Center of Marine Food, Key Laboratory of Advanced Processing of Aquatic Products of Guangdong Higher Education Institution, College of Continuing Education, Guangdong Ocean University, Zhanjiang, 524088, China; b Zhanjiang Institute for Food and Drug Control, Zhanjiang, 524022, China; c Department of Wine, Food and Molecular Biosciences, Lincoln University, Lincoln, Canterbury, 7647, New Zealand

**Keywords:** CdTe/CdS/SiO_2_, Quantum dots, "OFF-ON" sensor, Ammonium pyrrolidine dithiocarbamate, Cadmium ions

## Abstract

In this study, we have developed a novel fluorescent "OFF-ON" quantum dots (QDs) sensor based on CdTe/CdS/SiO_2_ cores. Ammonium pyrrolidine dithiocarbamate (APDC), ethylenediamine tetraacetic acid (EDTA), and 1,10-phenanthroline (Phen) served as potential chemical etchants. Among these three etchants, APDC exhibited the most pronounced quenching effect (94.06%). The APDC-etched CdTe/CdS/SiO_2_ QDs demonstrated excellent optical properties: the fluorescence of the APDC-etched CdTe/CdS/SiO_2_ QDs system (excitation wavelength: 365 nm and emission wavelength: 622 nm) was significantly and selectively restored upon the addition of cadmium ions (Cd^2+^) (89.22%), compared to 15 other metal ions. The linear response of the APDC-etched CdTe/CdS/SiO_2_ QDs was observed within the cadmium ion (Cd^2+^) concentration ranges of 0–20 μmol L^−1^ and 20–160 μmol L^−1^ under optimized conditions (APDC: 300 μmol L^−1^, pH: 7.0, reaction time: 10 min). The detection limit (LOD) of the APDC-etched CdTe/CdS/SiO_2_ QDs for Cd^2+^ was 0.3451 μmol L^−1^ in the range of 0–20 μmol L^−1^. The LOD achieved by the QDs in this study surpasses that of the majority of previously reported nanomaterials. The feasibility of using APDC-etched CdTe/CdS/SiO_2_ QDs for Cd^2+^ detection in seawater, freshwater, and milk samples was verified, with average recoveries of 95.27%–110.68%, 92%–106.47%, and 90.73%–111.60%, respectively, demonstrating satisfactory analytical precision (RSD ≤ 8.26).

## 1 Introduction

Cadmium (Cd), a representative heavy metal, poses a substantial threat to human health. Increasing human activities such as mining, smelting, industrial development, and agriculture release substantial quantities of Cd into the environment [1]. Cd contamination, especially in food, has elicited global concern [2]. The anthropogenic source of Cd is reported to be 0.002–0.008 mg L^−1^ (0.0178–0.0711 μmol L^−1^) in drinking water [3], with some food items like milk containing up to 0.27 μg L^−1^ (0.0024 μmol L^−1^) of Cd [4]. Unfortunately, even at low concentrations of 0.001–0.1 mg L^−1^ (0.0089–0.889 μmol L^−1^), Cd exhibits toxicity [5]. Therefore, the development of a rapid and sensitive sensor for Cd detection in water and food is imperative to ensure food safety.

Presently, methods for Cd determination include atomic absorption spectroscopy, atomic emission spectroscopy, inductively coupled plasma emission spectroscopy, inductively coupled plasma mass spectrometry, high-performance liquid chromatography, voltammetry, and colorimetry [[6], [7], [8], [9], [10], [11], [12]]. However, most Cd detection methods require prolonged analysis times, high equipment conditions, and complex operations that limit their practical application [13].

Fortunately, quantum dots (QDs), based on optical fluorescent responses, have paved the way for real-time Cd detection [14,15]. QDs are increasingly used for Cd detection owing to their rapid, easy usage and independence from complex instrumentation [[16], [17], [18], [19]]. However, quantum dots enhance aggregation tendencies due to their high surface activity resulting from their small size and large surface area. Additionally, the presence of defect states on the quantum dots' surface exacerbates the non-radiative recombination process, thereby negatively impacting the luminous efficiency of nanoparticles [20]. Additionally, their instability, low specificity, easy quenching, potential for leakage, and toxicity have hindered their broad applicability for Cd detection in water and food [21].

To overcome these disadvantages, this study developed a fluorescence quenching technology (OFF-ON) based on the recovery of QDs fluorescence quenching, facilitating the selective and rapidly detection of heavy metals [22,23]. Chelating agents, including ethylene diamine tetraacetic acid (EDTA), ammonium pyrrolidine dithiocarbamate (APDC), and 1,10-phenanthroline (Phen), have exhibited good fluorescence quenching effects when employed for QD etching [24]. Additionally, the core/shell/shell mode QDs have improved stability, reduced metal leakage, and lowered toxicity compared to single-core QDs [25,26]. Furthermore, the integration of a SiO_2_ shell into CdTe/CdS QDs has significantly enhanced their optical properties and biocompatibility within cellular environments [27,28].

**Fig. 1:**
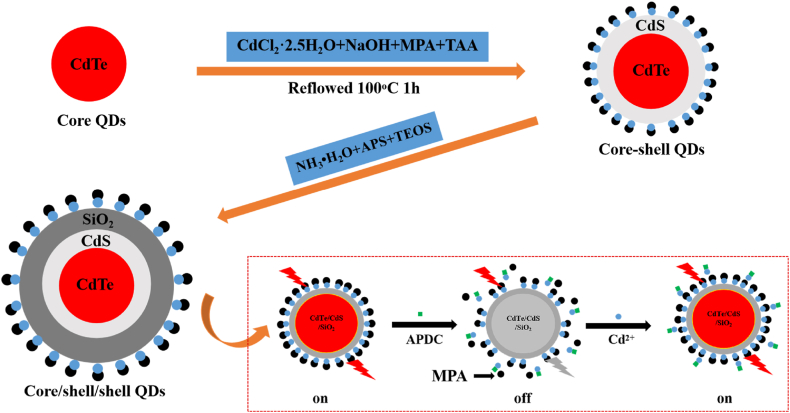
Schematic illustration of the preparation process of CdTe/CdS/SiO_2_ QDs and its the working principle for the visual fluorescence detection of Cd^2+^.

Based on these insights, this study has developed a novel CdTe/CdS/SiO_2_ QDs fluorescent nanomaterial for Cd^2+^ detection, utilizing CdS and SiO_2_ as shell components. EDTA, APDC, and Phen were employed as quenching agents to construct an “OFF-ON” CdTe/CdS/SiO_2_ QDs model. The operational principle of the developed sensor is depicted in Fig. 1. After the addition of APDC, its dithiocarbamate functional groups chemically corrode the surface of QDs. APDC can establish coordination bonds with Cd^2+^ present on the quantum dot's surface, disrupting the Cd-mercaptan layer and leading to the formation of Cd-APDC complexes [29]. This partial removal of the Cd-mercaptan layer reduces surface passivation, resulting in luminescence quenching. Upon the addition of Cd^2+^, a noticeable enhancement in fluorescence intensity was observed in the etched QDs. The coordination bonds between APDC and the Cd^2+^ ions on the quantum dot's surface may be disrupted or replaced by Cd^2+^ [30], causing the reappearance of Cd passivation layers on the QDs' surface, accompanied by the recovery of fluorescence intensity [31]. Using an ultraviolet lamp enables visual detection, leading to significant contributions towards the rapid and efficient detection of the Cd^2+^.

## 2 Experimental

### 2.1 Reagents

Tellurium powder (99%) was purchased from Hua Xia Chemical Reagent Co., LTD (Chengdu, China). Thioacetamide (TAA), Astragalus Polysaccharide (APS), Phen, and APDC were purchased from McLean. Tetraethyl orthosilicate (TEOS) and PBS were purchased from Sinopharm Group Chemical Reagent Co., LTD (Shanghai, China). EDTA was obtained from Zhanyun Chemical Co., LTD (Shanghai, China). CdCl_2_·2.5H_2_O (RON's reagent), 3-mercaptopropionic acid (MPA). K^+^, Na^+^, Ba^2+^, Mg^2+^, Ca^2+^, Hg^2+^, Fe^2+^, Fe^3+^, Pb^2+^, Zn^2+^, Cu^2+^, Al^3+^, Mn^2+^, Co^2+^, Ni^2+^ were derived from KCl, NaCl, BaCl_2_·2H_2_O, MgCl_2_, CaCl_2_, HgCl_2_, FeCl_3_, FeSO_4_·7H_2_O, Pb (NO_3_)_2_, ZnSO_4_·7H_2_O, CuSO_4_, Al (NO_3_)_3_·9H_2_O, MnSO_4_·H_2_O, CoCl_2_·6H_2_O, NiSO_4_·6H_2_O, respectively. All the reagents were used as received without further purification. Double-distilled water was used throughout this study.

### 2.2 Apparatus

The TEM image of the QDs was captured using a Transmission Electron Microscope (Talos F200X G2 TEM) (Thermo Fisher Scientific, USA) at 200 kV. A multifunctional enzyme spectrometer was used to measure UV–vis absorption and fluorescence spectra (Shimadzu, Japan). The infrared spectrum of the QDs was measured using a Fourier infrared spectrometer (FRIT) and the hydrated particle size was measured using a dynamic particle size scatterer (Nicomp N3000, USA). The pH was measured using a pH meter (Mettler-Toledo Instruments, China). All images were taken with a SONY digital camera (Alpha7 mark III, Japan) under 365 nm UV light.

### 2.3 Preparation of CdTe/CdS QDs

A mixture of 80 mg tellurium powder and 50 mg NaBH_4_ (dosage ratio 8:5) was prepared under N_2_ protection. The mixture was stirred until the solution became clear and transparent, forming NaHTe [32]. To prepare the Cd precursor, 0.1 mmol L^−1^ CdCl_2_-2.5H_2_O and 0.3 mmol L^−1^ mercaptopropionic acid (MPA) were dissolved in ultrapure water (100 mL) and adjusted to pH 8.5 with 0.5 mol L^−1^ NaOH. MPA serves as a prevalent surface ligand and stabilizer, binding to the CdTe core's surface and establishing a surface modification layer enriched with thiol functional groups [33]. The solution was deoxygenated with N_2_ for 1 h. Then, CdTe QDs were prepared by adding a freshly prepared NaHTe solution to the Cd precursor (Cd: Te: MPA = 2:1:4.8) and reflowing at 100 °C for 5 h. The core CdTe quantum dots are synthesized by thermally decomposing Cd precursors in the presence of a Te precursor. The CdTe QDs were washed several times with 2-propanol and dispersed in 5 mL of deionized water. The Cd precursor of the CdS shell was prepared by adding 0.1 mmol L^−1^ CdCl_2_-2.5H_2_O and 0.3 mmol L^−1^ MPA in 95 mL of ultrapure water, followed by pH adjustment to 8.5 with 0.5 mol L^−1^ NaOH. The growth of the CdS shell involved surface reactions on the CdTe core. Typically, TAA was employed as a sulfur source, supplying sulfur ions that facilitate the growth of CdS crystals [34]. After adding the Cd precursor of the CdS shell and 0.1 mmol L^−1^ TAA to the CdTe QDs solution, the solution mixture was reflowed at 100 °C for 1 h. During this process, Cd^2+^ on the surface of the CdTe core reacted with sulfur ions provided by TAA, resulting in the formation of the CdS crystal shell. Simultaneously, the thiol functional groups of MPA may coordinate with Cd^2+^ on the core surface, influencing the rate and morphology of shell growth [33]. The thiol functional groups of MPA also interacted with Cd^2+^ on the CdTe core surface, enhancing the stability of the quantum dots [35]. The prepared CdTe/CdS QDs were washed with 2-propanol and dispersed in 5 mL of ultrapure water.

### 2.4 Preparation of CdTe/CdS/SiO_2_ QDs hybrid probe

SiO_2_-coated CdTe/CdS (CdTe/CdS/SiO_2_) was synthesized according to the Stöber method [36]. In a typical synthesis procedure, the addition of 3 mL of water to the CdTe/CdS QDs ethanol solution resulted in the hydrolysis of the silica precursor. Subsequently, 1 mL of an aqueous ammonia solution was added to the mixture and the ammonia was used to catalyze the hydrolysis and condensation reactions [37]. The mixture was vigorously stirred, and then 15, 20, and 80 μL of APS and TEOS with a molar ratio of 2:1 was quickly injected. TEOS was commonly used as a silica precursor, and APS was employed to initiate the polymerization of silicon precursors. The samples were washed several times with ethanol and dissolved in ultrapure water for 20 min. When no precipitate was observed in the solution after standing for 24 h, CdTe/CdS/SiO_2_ was dispersed in 10 mL PBS by ultrafiltration (pH = 7.0) to obtain a concentration of 1 mg mL^−1^ of QDs.

### 2.5 Fluorescence quenching of CdTe/CdS/SiO_2_ QDs

The quenching abilities of EDTA, Phen, and APDC on the fluorescence of CdTe/CdS/SiO_2_ QDs were evaluated as previously described [[38], [39], [40]]. EDTA, Phen, and APDC solutions (1 mmol L^−1^, 30 μL) were individually added to the prepared 10 μL of the CdTe/CdS/SiO_2_ QDs suspension. The resulting CdTe/CdS/SiO_2_ QDs systems were diluted to a final volume of 100 μL using ultrapure water. Fluorescence spectrum of the CdTe/CdS/SiO_2_ QDs systems was measured using a multifunctional enzyme spectrometer (λex = 365 nm). The fluorescence quenching levels of the QDs systems were evaluated using ultrapure water as a blank sample because it was the diluent used in all tests. This study primarily focused on comparing these three etchants due to their well-established quenching abilities and their effectiveness in quenching QD fluorescence. By concentrating on comparing the fluorescence quenching levels induced by these three etchants on CdTe/CdS/SiO_2_, we aimed to avoid the inclusion of numerous other chemicals that could potentially distract our analysis. Through this focused comparison, we aimed to determine the optimal etchant for optimizing the design and performance of CdTe/CdS/SiO_2_ through a centralized approach.

### 2.6 Procedures of Cd^2+^ detection

Cd^2+^ was detected as described previously [41]. The reaction system for Cd^2+^ detection was prepared by mixing 10 μL CdTe/CdS/SiO_2_ QDs, 30 μL APDC (1 mmol L^−1^), and a fixed volume of Cd^2+^ in a series of micropores at room temperature. The mixture was diluted with PBS buffer (pH 7.0) to a final volume of 200 μL, and the corresponding photographs were recorded under UV irradiation in the dark (λex = 365 nm) to perform visual fluorescence detection. For spectroscopic analysis, the reactants' fluorescence spectra (λex = 365 nm) were recorded using a multifunctional enzyme-labeling device.

Under the optimal detection conditions, the fluorescence intensity of APDC/CdTe/CdS QDs solution samples without Cd^2+^ was determined, and the standard deviation was calculated from twenty repeated measurements. The limit of detection (LOD) was calculated by dividing three times the standard deviation by the slope of the calibration curve [31]. The LOD was calculated using equation (1):

(1) LOD=3δ/S

Where LOD is the limit of detection (δ is the standard deviation of the fluorescence intensity of APDC/CdTe/CdS QDs solution samples without Cd^2+^ and S is the slope of the calibration curve.

### 2.7 Optimal conditions (reaction dose, time, and pH) for the detection of Cd^2+^ by QDs-APDC

The effect of APDC concentration on the fluorescence quenching of CdTe/CdS/SiO_2_ QDs was investigated following established procedures [31]. Different volumes of 1 mmol L^−1^ APDC solutions were added to 10 μL of CdTe/CdS/SiO_2_ QD suspension. The resulting mixture was diluted with ultrapure water to a final volume of 100 μL to obtain a series of CdTe/CdS/SiO_2_-APDC mixed liquids with concentrations of 0, 10, 20, 30, 50, 70, 100, 200, 300, 500, and 900 μmol L^−1^. The fluorescence spectrum (λex = 365 nm) was measured using a multifunctional enzyme marker.

The effect of the Cd^2+^ concentration on the fluorescence recovery of the CdTe/CdS/SiO_2_-APDC system was evaluated as previously described [23]. 40 μL of CdTe/CdS/SiO_2_-APDC composite solution was mixed with various volumes of Cd^2+^ solutions (1 mmol L^−1^). The mixture was diluted with ultrapure water to a final volume of 100 μL to obtain a series of CdTe/CdS/SiO_2_-APDC-Cd^2+^ composite liquids with concentrations of 0, 10, 12, 14, 15, 16, 18, 20, 40, 100, 150, and 160 μmol L^−1^. Fluorescence spectra were measured using a multifunctional enzyme marker with an excitation wavelength of 365 nm.

The effect of reaction time on the fluorescence quenching of CdTe/CdS/SiO_2_ QDs (10 μL) induced by APDC (300 μmol L^−1^). The resulting mixture was diluted with ultrapure water to obtain a final volume of 100 μL. As per the typical procedure [42], the fluorescence intensity of CdTe/CdS/SiO_2_-APDC composite liquid (λex = 365 nm) was measured at 0, 1, 2, 4, 6, 8, 10, 15, and 20 min, respectively. The fluorescence quenching efficiency was calculated using equation (2):

(2) $$\text{Quenching}\;\text{efficiency}=\left(1-F/F_{0}\right)\times 100\%$$

where F/F_0_ is the relative fluorescence intensity ratio (F is the fluorescence intensity of the sensor system in the presence of APDC at different times and F_0_ is the fluorescence intensity of the QDs.

To determine the optimum detection time for the CdTe/CdS/SiO_2_-APDC system in detecting Cd^2+^ (150 μmol L^−1^), the resulting mixture was diluted with ultrapure water to a final volume of 100 μL [43]. The fluorescence intensity of CdTe/CdS/SiO_2_-APDC-Cd^2+^ composite solution (λex = 365 nm) was measured at 0, 1, 2, 4, 6, 8, 10, 15, 20, 25, and 30 min.

The interference of pH on the fluorescence intensity of the QDs suspension, CdTe/CdS/SiO_2_-APDC system, and CdTe/CdS/SiO_2_-APDC-Cd^2+^ system was investigated as previously described [42]. The preparation of the various systems followed the same procedure mentioned above. Experimental solutions were adjusted to different pH values (4, 5, 6, 7, 8, 9, and 10) by adding either 1 mol L^−1^ disodium phosphate or 0.1 mol L^−1^ NaOH. Fluorescence spectra (λex = 365 nm) were measured using a multifunctional enzyme marker at varying pH values.

### 2.8 Statistical analysis

All experiments were performed at least in triplicate, and the data are expressed as mean ± S.E. Data analyses were carried out using IBM SPSS Statistics 25. Data comparisons were performed using one-way ANOVA to compare the means of the different treatment groups. Statistical significance was set at P < 0.05.

## 3 Result and discussion

### 3.1 Characterization of the CdTe/CdS/SiO_2_ QDs hybrid probe

**Fig. 2:**
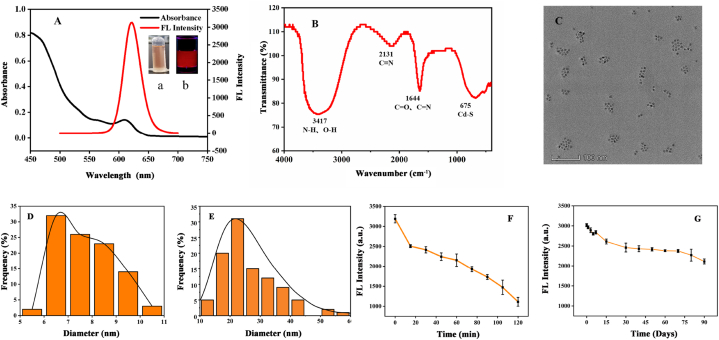
(A) UV–vis absorption spectra and fluorescence spectra (λex = 365 nm), inset: (a) Photographs of CdTe/CdS/SiO_2_ QDs in daylight and (b) under a UV lamp (λex = 365 nm). (B) FTIR of the CdTe/CdS/SiO_2_ QDs. (C) TEM image of CdTe/CdS/SiO_2_ QDs (D) The corresponding particle size distribution of CdTe/CdS/SiO_2_ QDs. (E) Hydration particle size distribution of CdTe/CdS/SiO_2_ QDs and the particle size of QDs in TEM image were calculated using Nano Measurer software. (F) The fluorescence intensity of CdTe/CdS/SiO_2_ QDs under a UV lamp (λex = 365 nm) was irradiated for 0, 15, 30, 45, 60, 75, 90, 105, and 120 min. (G) Fluorescence intensity of CdTe/CdS/SiO_2_ QDs stored at 4 °C for 0, 1, 3, 5, 7, 15, 30, 40, 50, 60, 70, 80, and 90 days.

In the full wavelength range (450–750 nm), the CdTe/CdS/SiO_2_ QDs exhibited an obvious maximum absorption peak at 608 nm and emitted intense fluorescence with a maximum emission wavelength of 622 nm (Fig. 2A). The QDs emitted a bright red fluorescence under ultraviolet (UV) light (365 nm).

FTIR spectra in the range of 400–4000 cm^−1^ were recorded, revealing a series of characteristic peaks (Fig. 2B). Among these peaks, the peak at 1647 cm^−1^ was assigned to the vibrations of the C=O and C=N group [44], indicating successful covalent bonding of MPA to the QDs’ surface [45]. The peak at 675 cm^−1^ can be attributed to the strong covalent bond between Cd and S [46]. The peak at 2131 cm^−1^ was attributed to the C≡N functional group [42]. The peak at 3417 cm^−1^ corresponds to the N–H or O–H functional group. The oxygen-containing functional groups in this study, such as the C=O bond and O–H bond, may enhance sensitivity by forming Cd^2+^ complexation [[47], [48], [49]].

The TEM image showed that the as-prepared QDs were either regular circles or ellipses and evenly distributed (Fig. 2C). 95% of the particle sizes were in the range of 6–10 nm, with an average particle size of 7.74 nm (Fig. 2D). The particle size for CdTe/CdS/SiO_2_ QDs is a crucial parameter that influences their performance in cadmium detection applications. Smaller QDs typically exhibit higher surface-to-volume ratios, leading to enhanced sensitivity due to increased interaction with Cd^2+^. The hydrated particle size was in the range of 15–40 nm (Fig. 2E). Fluorescence stability analysis showed that the fluorescence intensity of the QDs decreased after irradiation at 365 nm for 2 h (Fig. 2F). This phenomenon is typically followed by the stimulation of QDs by the light emission and changes in the internal lattice. Meanwhile, the energy level splitting within the QDs, the presence of surface defect states, and photon attenuation primarily occurred through non-radiative processes [50,51]. As shown in Fig. 2G, with the extension of storage time, the fluorescence intensity of the QDs decreased by only 30% for 90 days at 4 °C. These results signify the excellent fluorescence stability of the quantum dots (QDs). This stability can be attributed to the presence of a silica shell that acts as a protective barrier around the quantum dot core, effectively passivating surface defects and trap states. Such passivation leads to a reduction in non-radiative recombination of excitons, thereby enhancing the overall photoluminescence stability of the quantum dots [52]. Moreover, in comparison to previously studied quantum dots, the CdTe/CdS/SiO_2_ QDs designed in this study are coated with silicon using the Stober method. This synthesis technique is characterized by its simplicity, short preparation time, controllable particle size below 10 nm, and an impressive quantum yield of 87.26%. These attributes confer significant advantages for chemical detection applications.

### 3.2 APDC induced fluorescence quench of CdTe/CdS/SiO_2_ hybrid probe

**Fig. 3:**
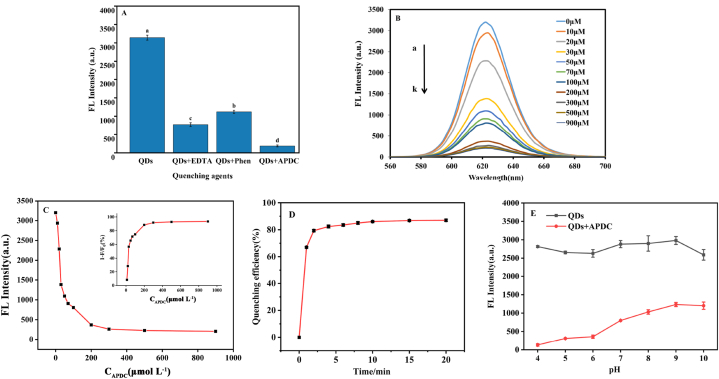
(A) Effect of etching on the fluorescence intensity of CdTe/CdS/SiO_2_. (B) Fluorescence spectra of CdTe/CdS/SiO_2_ upon the addition of different concentrations of APDC, from a to k: 0, 10, 20, 30, 50, 70, 100, 200, 300, 500, 700, and 900 μmol L^-1^. (C) Fluorescence intensity plots at 622 nm versus the APDC concentration. Inset: Plots of quenching efficiency at 622 nm versus APDC concentration. (D) Relationship between quenching efficiency (%) of APDC and time at 622 nm. (λex = 365 nm) (E) The effect of pH on the fluorescence intensity of QDs and QDs-APDC system at 622 nm.

To realize the OFF-ON function, EDTA, Phen, and APDC were selected as quenching agents for the QDs, and their fluorescence-quenching abilities were compared. Among these, APDC exhibited a superior fluorescence quenching effect (Fig. 3A). A Previous study has reported that APDC could effectively destroy the mercaptan layer of Cd and form a Cd-APDC complex, potentially leading to a partial loss of the mercaptan layer that may reduce the surface passivation of the QDs [53].

The effects of dosage, time, and pH on the quenching ability of APDC were evaluated. As shown in Fig. 3B, the fluorescence intensity of the QDs gradually decreased with increasing concentrations of APDC. The quenching efficiency of APDC raised to 94.06% at a concentration of 300 μmol L^−1^ (Fig. 3C). This result is similar to an earlier report in which the quenching efficiency of APDC for 0.5 μmol L^−1^ CdTe/CdS QDs reached a maximum at 15 μmol L^−1^ [54].

We further assessed the impact of reaction time on the quenching efficiency of APDC for QDs. As shown in Fig. 3D, the quenching efficiency of APDC rapidly increased to 66.96% after 1 min of APDC addition. The quenching efficiency further increased with increasing reaction time and remained at a high level after 10 min. The highest efficiency is achieved after 10 min.

For the pH stability assessment, the fluorescence spectrum of QDs solutions was monitored at various pH values (Fig. 3E). The experimental samples were prepared by dispersing QDs in solutions of various pH (4–10). While the pH was gradually increased from 4 to 10, the fluorescence intensity of the QDs remained high. However, the quenching efficiency of APDC notably declined with increasing pH, indicating a significant pH-dependent effect on the QDs-APDC system. This observation aligns with the findings of Zhang et al. [55], who reported effective chelation of Cd on QDs surfaces by APDC at pH 4.0.

### 3.3 The feasibility of QDs-APDC system in detecting Cd^2+^

**Fig. 4:**
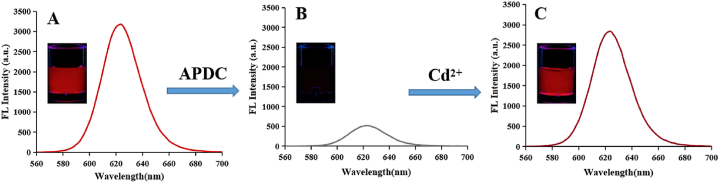
Fluorescence spectra and corresponding photographs of (A) CdTe/CdS/SiO_2_. (B) CdTe/CdS/SiO_2_-APDC (300 μmol L^-1^). (C) CdTe/CdS/SiO_2_-APDC-Cd^2+^ (150 μmol L^-1^), with λex = 365 nm.

To verify the OFF-ON function, the fluorescence intensity values of QDs-APDC before and after Cd^2+^ exposure were compared (Fig. 4). A reduction in the fluorescence intensity of the QDs was observed upon the addition of 300 μmol L^−1^ of APDC (Fig. 4A and B), indicating that QDs-APDC system was in the OFF state. After adding 150 μmol L^−1^ of Cd^2+^, the fluorescence intensity of the QDs-APDC-Cd^2+^ system was restored (Fig. 4C). This result suggested that Cd^2+^ could reactivate the QDs-APDC system by reforming the Cd–thiol complex to repair the destroyed surface of the QDs.

### 3.4 Selectivity and anti-interference of QDs-APDC in the detection of Cd^2+^

**Fig. 5:**
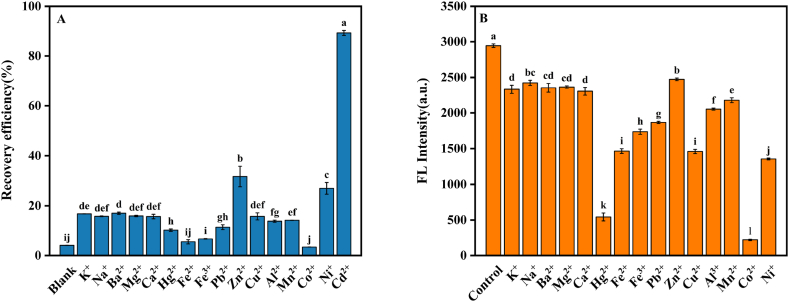
(A) The recovered fluorescence intensity of the QDs-APDC system in the presence of Cd^2+^ and other metal ions (150 μmol L^-1^). (B) Effect of Cd^2+^ on fluorescence recovery of the QDs-APDC system under interference from other coexisting ions.

To assess the selectivity of the QDs-APDC for detecting Cd^2+^, the responses of QDs-APDC to various metals, including K^+^, Na^+^, Ba^2+^, Mg^2+^, Ca^2+^, Hg^2+^, Fe^2+^, Fe^3+^, Pb^2+^, Zn^2+^, Cu^2+^, Al^3+^, Mn^2+^, Co^2+^, Ni^2+^, and Cd^2+^, were recorded. The results showed that the QDs-APDC exhibited a significantly greater response to Cd^2+^ (with the highest recovery efficiency) compared to its response to other metals (Fig. 5A). QDs-APDC exhibited an excellent selectivity toward Cd^2+^. CdTe/CdS/SiO_2_ QDs were suitable for sensitive detection, due to the high fluorescence intensity and narrow emission spectrum of CdTe core, and the addition of CdS shell can further improve the stability and quantum yield of quantum dots. This can also be attributed to the stronger affinity of Cd^2+^ for sulfhydryl groups on the surface of QDs capped with MPA [[56], [57], [58]], This affinity is sufficient to break the complexation of APDC with Cd atoms on the surface of QDs. QDs-APDC also showed a minor response to the Zn^2+^, which may be attributed to the similar electron configuration and chemical properties between Zn^2+^ and Cd^2+^ [[59], [60], [61]].

In addition, we explored the anti-interference properties of the APDC-QDs system with other metals in the detection of Cd^2+^. As shown in Fig. 5B, Hg^2+^ and Co^2+^ exhibited apparent interference with the APDC-QDs system in the detection of Cd^2+^. This result is similar to an earlier report in which Hg^2+^ interfered with the detection of Cd^2+^ by APDC/CdTe/CdS QDs. This interference by Hg^2+^ and Co^2+^ could be attributed to their stronger ability to bind S to mercaptan than that of Cd^2+^. It is also possible that the solubilities of CoTe and HgTe are lower than those of CdTe [31]. Consequently, when Hg^2+^ and Co^2+^ were added, CoTe and HgTe accumulated on the surface of the QD, which hindered the filling of holes by Cd^2+^, resulting in a reduced degree of recovery of the fluorescence intensity of the APDC-QDs system.

### 3.5 Optimal conditions for the detection of Cd^2+^ by QDs-APDC

**Fig. 6:**
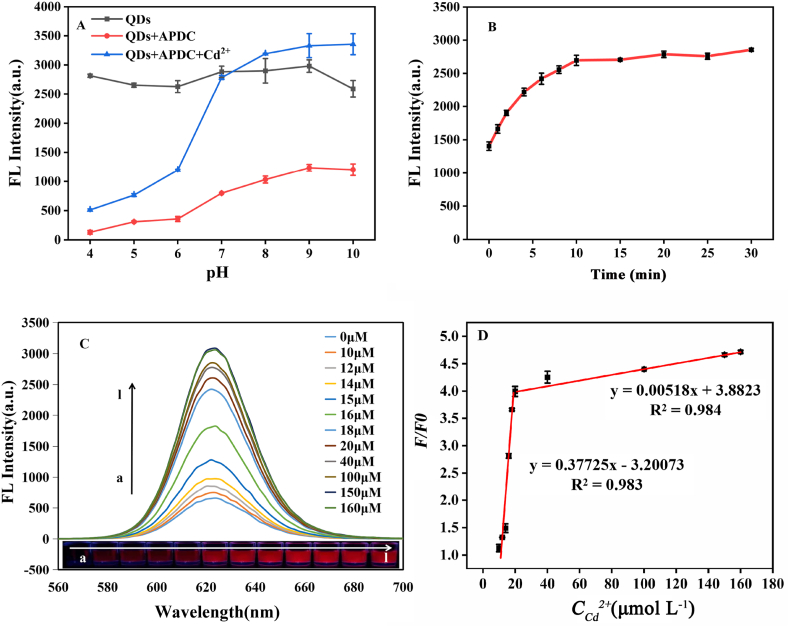
(A) Effect of pH on the fluorescence intensity of the QDs, QDs-APDC system, and QDs-APDC-Cd^2+^ system at 622 nm. (B) Fluorescence intensity of QDs-APDC at 622 nm as a function of time. (C) Fluorescence spectra and the corresponding photographs under the UV lamp of the nanosensor upon exposure to different concentrations of Cd^2+^ (0, 10, 12, 14, 15, 16, 18, 20, 40, 100, 150, and 160 μmol L^-1^). (D) Linear relationship between F/F_0_ and concentration of Cd^2+^. (λex = 365 nm).

To optimize the pH condition for Cd^2+^detection by QDs-APDC, the fluorescence response of QDs-APDC to Cd under various pH conditions was assessed. As shown in Fig. 6A, the fluorescence response of QDs-APDC to Cd^2+^ (Bule) was suppressed at low pH (4–6). However, this suppression by pH was weakened and disappeared at higher pH (7–10).

To determine the optimal reaction time for Cd^2+^ detection by QDs-APDC, the fluorescence (FL) intensity of the QDs-APDC system in the presence of Cd^2+^ was recorded at different reaction times. The results showed that the FL intensity first declined and then stabilized after 10 min of reaction (Fig. 6B). Therefore, 10 min was determined as the optimal reaction time for Cd^2+^ detection by QDs-APDC.

### 3.6 Analytical performance of the QDs-APDC system

To evaluate the analytical performance of the QDs-APDC system in detecting Cd^2+^, the dose-response relationship between the FL intensity of QDs-APDC and the Cd^2+^ concentration was measured. As shown in Fig. 6C, the FL intensity of QDs-APDC gradually increased with an elevation in the Cd^2+^ exposure concentration, accompanied by a slight redshift. According to the values (F/F_0_) of the fluorescence intensity of QDs-APDC in the presence of different concentrations of Cd^2+^, two linear dose-response relationships were observed in the ranges of 0–20 μmol L^−1^ and 20–160 μmol L^−1^ (Fig. 6D). Equation *F/F*_*0*_ = −3.20073 + 0.37725*C*_*Cd*_^*2+*^*,* with a correlation coefficient of 0.983, was obtained for the range of 0–20 μmol L^−1^ of Cd^2+^. Another equation, *F/F*_*0*_ = 3.8823 + 0.00518*C*_*Cd*_^2+^, with a correlation coefficient of 0.984, was obtained for the range of 20–160 μmol L^−1^ of Cd^2+^. Where F and F_0_ are the fluorescence intensity of the sensing system in the presence and absence of Cd^2+^, respectively.

Under the optimal detection conditions, the standard deviation and the slope of the calibration curve (0–20 μmol L^−1^) (Fig. 6D) were substituted into equation (1) to calculate the LOD of the QDs-APDC for Cd^2+^ detection, yielding a value of 0.3451 μmol L^−1^. The limit of quantitation (LOQ) of the QDs-APDC for Cd^2+^ detection was 1.150 μmol L^−1^. Compared with different QD sensors for Cd^2+^ detection, as previously reported [62], our QDs-APDC had a lower LOD and higher sensitivity. Table 1 is the comparison of different QD sensors for Cd^2+^ determination.

**Table 1:** Comparison of different QD sensors/fluorescent probes for Cd^2+^ determination.

QD Sensor	Linear Range (μmol L^−1^)	LOD (μmol L^−1^)	Reference
QDs@mSiO_2_-RB	0–80	1.25	[63]
S^2−^ modified CdTe QDs	1.3–25	0.5	[64]
Ag_2_S QD	1–40	0.5460	[62]
CQDs and CdTe QDs	0.1–23	0.018	[65]
N, S co-doped carbon quantum dots/Au nanoclusters	0–2.1	0.062	[66]
CdTe/CdS/SiO_2_ QD	0–20	0.3451	This work

### 3.7 Analytical applications

The accuracy of QDs-APDC for Cd^2+^ detection was assessed by measuring the recovery ratio of Cd^2+^ and the relative standard deviation (RSD) of samples. In the pretreatment stage, known concentrations of Cd^2+^ (10, 20, and 40 μmol L^−1^) were added to seawater (Pacific Ocean), freshwater (Huguang Lake), and milk (whole milk). The results showed that the recovery ratios of Cd^2+^ in seawater, freshwater, and milk were 95.27%–110.68%, 92.00%–106.47%, 90.73%–111.60%, respectively, and RSD ≤ 8.26% (Table 2). The larger RSD observed in seawater compared to freshwater may be attributed to the complex components present in seawater, such as chloride, carbonate, and fluoride salt soluble components [67]. Compared with the QDs developed by Zhou [68], QDs-APDC exhibited higher average recovery in detecting Cd^2+^ in seawater. Its average recovery in detecting Cd^2+^ is also higher than that of other QDs in lake water [69], and even in milk [70]. These results indicated that QDs-APDC can be used to detect Cd^2+^ in water (seawater, freshwater) and milk samples.

**Table 2:** Application of the proposed nanosensor in seawater, freshwater, and milk samples (n = 3).

Samples	Added (μmol L^−1^)	Detected (μmol L^−1^)	RSD (%)	Recovery (%)
Seawater 1	10	11.07	4.48	110.68
Seawater 2	20	19.05	8.26	95.27
Seawater 3	40	38.82	6.30	97.06
Freshwater 1	10	10.65	4.28	106.47
Freshwater 2	20	18.40	5.78	92.00
Freshwater 3	40	39.82	6.61	99.55
Milk 1	10	11.16	4.78	111.60
Milk 2	20	18.15	6.84	90.73
Milk 3	40	40.36	5.65	100.91

## 4 Conclusion

In this study, we conducted a comparative analysis of the quenching effects of EDTA, Phen, and APDC on CdTe/CdS/SiO_2_ QDs. The result revealed that APDC exhibited the most potent quenching effect (94.06%). The comparison provided critical information for selecting the most effective quencher and improving the overall performance of sensing technologies. Specifically, the APDC-etched CdTe/CdS/SiO_2_ QDs displayed effective and selective restoration in the presence of Cd^2+^, comparing to 15 other metal ions. By optimizing parameters such as APDC concentration, pH, and reaction time, we successfully developed a novel "OFF–ON" QDs-APDC system for Cd^2+^ detection. The QDs-APDC system exhibited a relatively low LOD (0.3451 μmol L^−1^) and displayed two linear dose-response ranges were 0–20 μmol L^−1^ and 20–160 μmol L^−1^. Importantly, the "OFF-ON" mechanism enabled real-time detection, with fluorescence changes occurring immediately upon Cd^2+^ binding, facilitating rapid results acquisition. Furthermore, the QDs-APDC system exhibited favorable RSD values and demonstrated high average recovery rates when applied to the detection of Cd^2+^ in seawater, freshwater, and milk samples. Consequently, QDs-APDC based on a CdTe/CdS/SiO_2_ core has potential application value in the rapid monitoring of Cd in the environment and food.

## Data availability

Data will be made available on request.

## CRediT authorship contribution statement

**Jiaqian Chen:** Writing – review & editing, Writing – original draft, Validation, Methodology, Investigation, Formal analysis, Conceptualization. **Haimei Meng:** Writing – review & editing, Writing – original draft, Methodology, Investigation, Formal analysis. **Zhijia Fang:** Writing – review & editing, Supervision, Resources, Project administration, Funding acquisition. **Iddrisu Lukman:** Writing – review & editing. **Jialong Gao:** Resources, Methodology, Formal analysis. **Jianmeng Liao:** Validation, Resources. **Qi Deng:** Resources, Data curation, Conceptualization. **Lijun Sun:** Validation, Resources, Methodology. **Ravi Gooneratne:** Writing – review & editing, Resources.

## Declaration of competing interest

The authors declare that they have no known competing financial interests or personal relationships that could have appeared to influence the work reported in this paper.
